# Supplementary material for: Aromatic Characterization of Mangoes (Mangifera indica L.) Using Solid Phase Extraction Coupled with Gas Chromatography–Mass Spectrometry and Olfactometry and Sensory Analyses
Source: Foods. 2020 Jan 9;9(1):75. doi: 10.3390/foods9010075 (PMC7023010; doi:10.3390/foods9010075)
Supplement: Supplementary file 1 [file foods-09-00075-s001.pdf]

Table S1. Physicochemical characterization of five different cultivars of mango samples

Cultivars	JM	QM	GF	HY	TN
TA(g/100g)	4.96±0.02[e]	8.20±0.01[b]	6.93±0.02[d]	9.01±0.01[a]	7.30±0.01[c]
TSS (ºBrix)	12.38±0.12[e]	14.81±0.01[d]	16.02±0.20[c]	17.91±0.03[a]	16.54±0.12[b]
pH	4.99±0.02[a]	4.66±0.01[c]	4.86±0.02[b]	4.50±0.01[d]	4.82±0.02[b]
L	50.38±0.13[c]	49.74±0.07[b]	51.44±0.05[a]	51.70±0.03[d]	61.76±0.14[cd]
a	4.64±0.02[c]	-0.67±0.06[b]	4.80±0.02[b]	5.10±0.07[c]	6.56±0.11[a]
b	38.51±0.04[a]	30.64±0.06[b]	36.88±0.08[e]	40.57±0.04[c]	55.61±0.12[d]
Total Sugar content (g/g d.w.)	8.43±0.05[e]	10.18±0.25[d]	14.81±0.07[c]	17.23±0.22[a]	14.97±0.12[b]
Reducing sugar content (g/g d.w)	5.84±0.26[c]	3.83±0.08[e]	5.09±0.01[d]	6.66±0.03[b]	7.17±0.21[a]
β-carotene (mg/g d.w.)	0.44±0.08[b]	0.28±0.02[d]	0.28±0.04[e]	0.32±0.02[c]	0.81±0.01[a]
TPC (mg GAE/g d.w)	18.05±0.05[a]	10.84±0.55[c]	14.48±0.09[e]	10.83±0.05[b]	25.43±0.52[d]
DPPH Scavenging activity (mg Vc/100g)	1.86±0.12[e]	0.74±0.08[c]	1.65±0.06[d]	1.07±0.04[b]	2.46±0.11[a]

JM: Jinmang cultivar; QM: Qingmang cultivar； GF: Guifei cultivar; HY: Hongyu cultivar; TN: Tainong cultivar. The mean and standard deviation (n=3) were calculated for three replicates. Values in total data with different letters are significantly different ($p < 0.05$).

Table S2. Statistical analysis for flavor attributes of five varieties mango samples.

Cultivars	JM	QM	GF	HY	TN
overall aroma	1.85±0.88[d]	1.55±0.44[e]	2.00±0.66[c]	2.10±0.55[b]	2.4±0.39[a]
tropical fruit	1.75±0.75[c]	1.8±0.67[b]	1.8±0.48[b]	1.85±0.41[a]	1.75±0.35[c]
floral	1.35±0.82[d]	1.40±0.61[c]	1.55±0.69[a]	1.45±0.64[b]	1.2±0.42[e]
honey/sweet	1.7±0.54[c]	1.4±0.7[d]	1.95±0.57[a]	1.85±0.67[b]	1.65±0.41[c]
green	0.65±0.34[d]	0.85±0.53[c]	0.95±0.55[b]	0.65±0.47[d]	1.45±0.55[a]
melon	0.9±0.52[d]	1.4±0.66[b]	1.55±0.55[a]	1.15±0.24[c]	1.55±0.6[a]
wood	1.1±0.21[d]	1.1±0.52[d]	1.4±0.52[b]	1.2±0.42[c]	1.6±0.52[a]
rosin	1.5±0.47[b]	1.4±0.7[c]	1.3±0.54[d]	1.1±0.46[e]	1.75±0.35[a]

JM: Jinmang cultivar; QM: Qingmang cultivar; GF: Guifei cultivar; HY: Hongyu cultivar; TN: Tainong cultivar. The mean and standard deviation (n=10) were calculated for three replicates. Values in total data with different letters are significantly different ($p < 0.05$)
